# Large asymptomatic Left Atrial Myxoma with ossification: case report

**DOI:** 10.1186/1749-8090-3-19

**Published:** 2008-04-29

**Authors:** Mattheos Panagiotou, Nikolaos D Panagopoulos, Panagiota Ravazoula, Loukas Kaklamanis, Efstratios N Koletsis

**Affiliations:** 1 Department of Cardiac Surgery, Athens Medical Center, Athens Greece; 2 Department of Cardiothoracic Surgery, School of Medicine, University of Patras, Greece; 3 Department of Pathology, School of Medicine, University of Patras, Greece; 4 Department of Pathology, Onassis Cardiac Surgery Center, Athens, Greece

## Abstract

**Background:**

Atrial myxomas are the most common primary cardiac tumors. They are usually small or moderate in size by the time of the diagnosis, exhibiting non specific cardiac or systemic symptoms, and are most frequently soft and friable without microscopic signs of ossification. We describe herein an extremely rare case of an asymptomatic giant left atrial myxoma with angiographic neovascularization and ossification.

**Case presentation:**

An asymptomatic 58-year-old male with a giant left atrial tumor, was transferred to our Unit for surgical treatment. The tumor was an incidental finding during a work-up for hemoptysis due to bronchectasis. The coronary angiogram showed tumor vessels originating from the RCA. The tumor macroscopically did not resemble a myxoma, considering its dimensions (12 × 10 cm) and its solid substance. The mass was excised together with the interatrial septum and the right lateral LA wall close to the right pulmonary veins orifices. The defect was closed with Dacron patches in order to prevent malformation of both atria. The pathology study revealed a benign myxoma with excessive osteoid (mature bone) content.

**Conclusion:**

We consider our case as extremely rare because of the asymptomatic course despite the large size of the tumor, the blood supply from the right coronary artery and the bone formation.

## Background

Atrial myxomas are the most common primary cardiac tumors. They are usually small or moderate in size by the time of the diagnosis, exhibiting non specific cardiac or systemic symptoms, and are most frequently soft and friable without microscopic signs of ossification. We describe herein an extremely rare case of an asymptomatic giant left atrial myxoma with angiographic neovascularisation and mature bone elements.

## Case presentation

**Figure 1:**
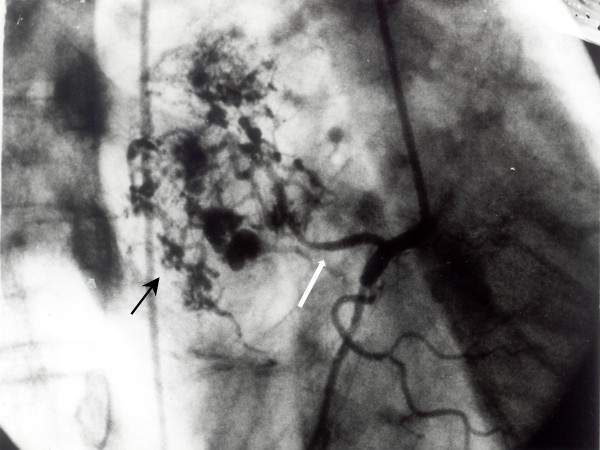
**Coronary angiography**. Coronary angiography, white arrow denote major atrial branch of the right coronary artery supplying the left atrial tumor mass, marked neovascularity with clusters of tortuous vessels, and blood pooling (light arrow).

**Figure 2:**
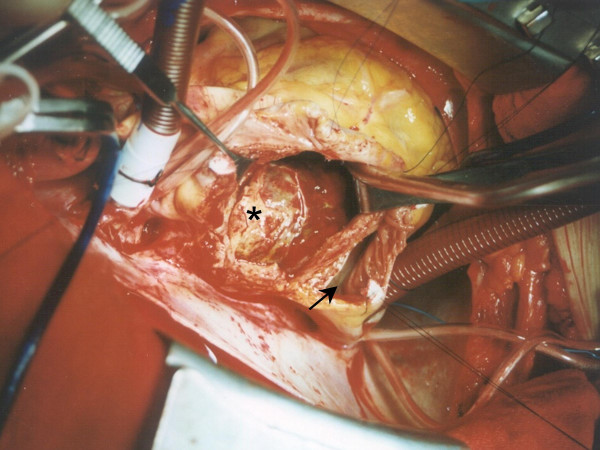
**Intraoperative picture**. Intraoperative picture of the mass (asterisk). Light black arrow denotes right atrium.

**Figure 3:**
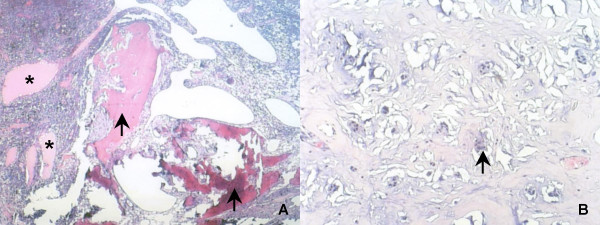
**Histopathological picture**. **Panel A**: Solid sheets of polygonal cells, thin-walled vessels containing proteinaseous material (asterisk) and a foci of ossification (light arrow) are observed in this figure (H/E 10×). **Panel B**: New bone formation. Note osteoblasts (light arrow) (H/E 40×).

A 58-year-old male was referred for evaluation of a calcified left atrial mass that was found in a thoracic CT scan performed during the course of haemoptysis investigation and was attributed to lung bronchiectasis after pulmonary work-up. Patient's past medical history was unremarkable. Further evaluation with transesophageal echocardiography revealed a sessile lesion measuring 10 × 12 cm in diameter arising from the interatrial septum in an otherwise normal-sized left atrium. Coronary angiography followed showing well organized tumor vessels arising from the right coronary artery (fig. 1). The tumor macroscopically did not resemble a myxoma considering its dimensions and its solid substance (fig. 2). The macroscopic appearance of the lesion intraoperatively was a soft, glistening, multi-lobulated mass with pale gray-white surface and a broad base attached near the fossa ovalis of the left atrium by a stalk. The mass was excised together with the interatrial septum and the right lateral LA wall close to the right pulmonary veins orifices. The defect was closed with Dacron patches in order to prevent malformation of both atria. Areas of hemorrhages were also present. Histopathological study revealed a benign myxoma with round, polygonal, or stellate cells surrounded by myxoiod stroma. Areas of hemorrhage, engorged arteries, veins and lymph vessels but most surprisingly areas of metaplastic bone tissue at the base of the lesion were also noted. No signs of malignancy were observed (Fig. 3). The patient tolerated the procedure well. His postoperative course was uneventful except the need for temporary external pacing. He was discharged on the 10^th ^postoperative day in sinus rhythm and remains well and asymptomatic two years after surgery.

## Conclusion

Atrial myxomas are benign slow growing neoplasms that arise from the interatrial septum and extend into the left or right atrium. Usually they are associated with atypical symptoms and only a 4% remain completely asymptomatic [1]. Clinical manifestations are in close relation to the location site and produced by mechanical interference with cardiac function or by intracardiac obstruction. Left-sided myxomas may present with signs of mitral stenosis or insufficiency. Embolization of systemic or pulmonary circulation is a frequent phenomenon [2]. and is observed in about 30–40% of patients. Echocardiography, especially TEE, is the method of choice to differentiate among intra- or peri-cardial mass lesions attached to the cardiac wall and furthermore may assess the characteristics of echocardiographic morphology of cardiac myxoma and its relation to systemic embolization [1,3].

Coronary angiography a useful diagnostic tool for evaluating tumor vascularity and rule out concomitant coronary artery disease should be performed in all patients over 40 years of age. Neovascularization has been reported up to 80% of myxoma cases [4]. Reynen demonstrated extensive neovascularity in 9 out of 37 patients who underwent coronary angiography as preoperative workup before atrial myxoma surgery [5]. Moreover it has been described that left-sided myxomas, are mostly supplied by the left circumflex artery [6]. This was not observed in our case, since blood supply of the tumor was provided solely by the right coronary [7]. It is known that calcification is present in 10–20% of myxomas, but bone formation has been reported only in a 68-year-old female by Ishikawa et al [8] and in a 6-year-old domestic shorthair cat [9]. A case report with a combination of left atrial myxoma with ossification and extra-medullary hematopoiesis has been reported by Kugai [10].

We consider our case as extremely rare because of the asymptomatic course despite the large size of the tumor, the blood supply from the right coronary artery and the bone formation.

## Competing interests

The authors declare that they have no competing interests.

## Authors' contributions

All authors have made substantial contributions to conception and design, or acquisition of data, or analysis and interpretation of data and have been involved in drafting the manuscript or revising it critically for important intellectual content. All authors read and approved the final manuscript.

MP: Manuscript Preparation, Literature Search, Study Design.

NP: Manuscript Preparation, Literature Search.

PR: Histo-pathologic diagnosis, Data Interpretation, Literature Search.

LK: Histo-pathologic diagnosis, Data Interpretation, Literature Search.

EK: Manuscript Preparation, Literature Search.
